# The role of repetition and reinforcement in school-based oral health education-a cluster randomized controlled trial

**DOI:** 10.1186/s12889-015-2676-3

**Published:** 2016-01-04

**Authors:** Abdul Haleem, Muhammad Khalil Khan, Shamta Sufia, Saima Chaudhry, Muhammad Irfanullah Siddiqui, Ayyaz Ali Khan

**Affiliations:** 1 Department of Oral Health Sciences, Federal Postgraduate Medical Institute, Shaikh Zayed Medical Complex, Lahore, 54600 Pakistan; 2 Department of Community and Preventive Dentistry, de’ Montmorency College of Dentistry, Fort Road, Lahore, Pakistan; 3 Department of Oral Pathology, University of Health Sciences, Lahore, Pakistan; 4 Department of Community Medicine and Pilgrims, Umm Al-Qura University, Makkah Al-Mukarrama-7607, Mecca, Saudi Arabia

**Keywords:** Repeated and reinforced oral health education, Dental health education, School-based, Dentist-led, Teacher-led, Peer-led

## Abstract

**Background:**

Repetition and reinforcement have been shown to play a crucial role in the sustainability of the effect of Oral Health Education (OHE) programs. However, its relevance to school-based OHE imparted by different personnel is not depicted by the existing dental literature. The present study was undertaken to determine the effectiveness of the repeated and reinforced OHE (RR-OHE) compared to one-time OHE intervention and to assess its role in school-based OHE imparted by dentist, teachers and peers.

**Methods:**

The study was a cluster randomized controlled trial that involved 935 adolescents aged 10-11 years. Twenty four boys’ and girls’ schools selected at random in two towns of Karachi, Pakistan were randomly assigned to three groups to receive OHE by dentist (DL), teachers (TL) and peer-leaders (PL). The groups received a single OHE session and were evaluated post-intervention and 6 months after. The three groups were then exposed to OHE for 6 months followed by 1 year of no OHE activity. Two further evaluations at 6-month and 12-month intervals were conducted. The data were collected by a self-administered questionnaire preceded by a structured interview and followed by oral examination of participants.

**Results:**

The adolescents’ oral health knowledge (OHK) in the DL and PL groups increased significantly by a single OHE session compared to their baseline knowledge (*p* < 0.05) and the increase was sustained over 6 months. Although one-time OHE resulted in a significant improvement in adolescents’ oral health behavior (OHB) related to the prevention of gingivitis in the two groups (*p* < 0.05), no significant change was observed in their behavior towards prevention of oral cancer. One-time teacher-led OHE was ineffective in improving adolescents’ OHK and OHB. The oral hygiene status (OHS) of the participants in all three groups did not change statistically after one-time OHE. The OHK, OHB and OHS indices increased significantly 6 months after RR-OHE than the initial scores (*p* < 0.001) irrespective of OHE strategy. Although the OHK scores of the DL and PL groups decreased significantly at 12-month evaluation of RR-OHE (*p* < 0.05), the said score of the TL group; and OHB and OHS scores of all three groups remained statistically unchanged during this period.

**Conclusions:**

The repetition and reinforcement play a key role in school-based OHE irrespective of educators. The trained teachers and peers can play a complementary role in RR-OHE.

## Background

Repetition and reinforcement have been shown to play a crucial role in the sustainability of health behavior [1]. According to Hartley [2] repetition and reinforcement are two of the four key principles of learning, the other two being clarity of objectives and active involvement of learner. Repetition and reinforcement appear to be mutually supportive. On one hand reinforcement has been shown to increase the likelihood that a newly learned behavior will be repeated in future [3] and on the other hand repetition helps in reinforcing the health education messages [4]. Previous reviews of oral health education suggest that OHE programs can have only short term gain in oral health behavior and oral health status [5–7]. This is especially true for most of the single session OHE programs [8–13]. Flanders found long term educational programs comparatively more effective than the ones based on short term interventions [14]. OHE programs have been shown to produce changes in knowledge, attitudes and behavior of the participants. However, different children are ready for the change at different times [15]. Hence, oral health education should be a continuous activity [12]. OHE programs incorporating repetition have generally been shown to be effective [16–19]. On the contrary some well designed programs of DHE for adolescents like ‘Natural Nashers’ were able to maintain positive oral health attitudes and behavior over 6 months without additional reinforcement [20].

A material reward [21] and an appreciation of a changed or newly adopted behavior [22–24] have been common means of reinforcement in OHE. Some studies have also utilized self-examination as a reinforcement tool [25–27]. The repetition and reinforcement have, therefore, a substantial role to play in the sustainability of the effect of OHE programs. However, the evidence for their relevance to various educator-led strategies [28] of school-based OHE is lacking in the existing dental literature. The present study was conducted to determine the effectiveness of the repeated and reinforced OHE (RR-OHE) compared to one-time OHE intervention, and to assess its role in dentist-led, teacher-led and peer-led strategies of school-based OHE.

## Methods

The present study was a cluster randomized controlled trial following a parallel design. It involved three groups of adolescents, each group receiving OHE either by dentist (dentist-led, DL), teachers (teacher-led, TL) or peer leaders (peer-led, PL). The DL group was used as a positive control. The trial was approved by the Institutional Review Board of Shaikh Zayed Medical Complex, Lahore (Ref. No. SZH/IRB/017-03) and was registered with the Current Controlled Trials (http:www.controlled-trials.com/isrctn) under the ISRCTN number39391017. It started on 1st January 2004 and continued till 28th February 2006. The trial was conducted in two towns of the cosmopolitan city of Karachi, Pakistan. The towns, having an overwhelming majority of Urdu-speaking people (Muhajirs), were chosen because of their ethnic homogeneity [29].

A detailed methodology pertaining to sample selection, randomization of the study groups, OHE intervention, follow up, selection and training of educators, process evaluation, data collection methods (questionnaire study and oral examination), blinding, and data organization and analysis has been published previously [28].

### Study population

The study population comprised of school children aged 10–11 years studying in class six of public and private schools of the two selected towns. The group of children in a section of class six in each school was considered a cluster.

### Sample size

The required number of participants in each study group was calculated as 327 to achieve 80 % power of the study at an α level of 0.05 [30, 31] based on the assumption that the OHE interventions understudy would produce a 50 % reduction in the existing prevalence of gingivitis (34 %) in 12 years old urban school children in Pakistan [32]. The number of clusters in each study group was determined as eight with 35–45 students in each cluster [33].

### Selection and randomization of study groups

**Fig. 1:**
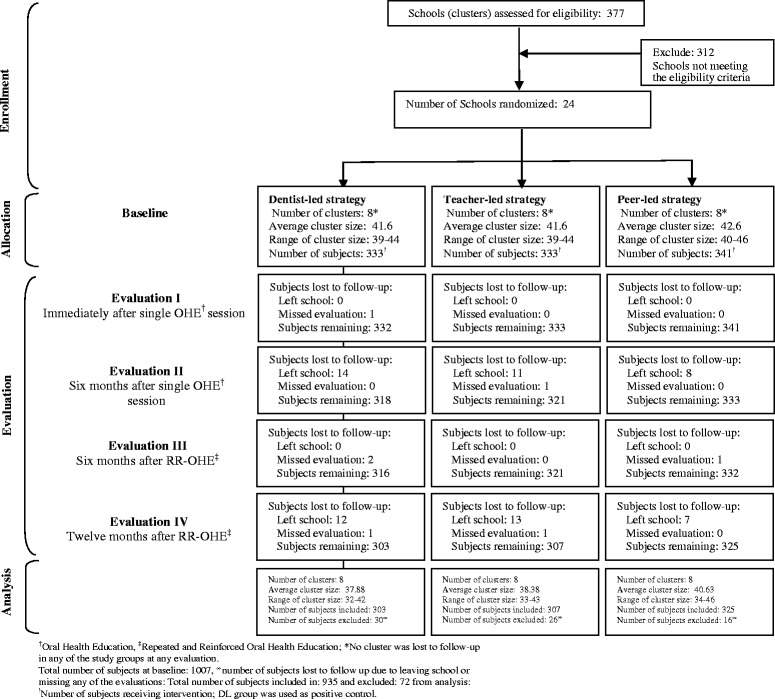
Flow chart of clusters and study subjects through different phases of the trial

The boys’ and girls’ schools in public and private categories having more than one section of class six and a minimum of 35 students per section were eligible to participate. A total of 377 public and private schools listed by the Education Department, Government of Sindh were assessed for eligibility (Fig. 1). Public schools (*n* = 124) had distinct categories of boys’ (*n* = 75) and girls’ (*n* = 49) schools but all private schools (*n* = 253) had co-education. A total of 65 schools met the eligibility criteria. From amongst the eligible schools, 12 girls’ and 12 boys’ sections were randomly chosen for the study. Two boys’ and two girls’ sections each from amongst the selected public and private schools were randomly allocated to each of the three study groups by a teacher, not involved in the study, using a lottery method.

The parents/ guardians of all children involved in the project were sent introductory letters and the consent forms through the offices of the school principals. The letter mentioned some benefits the participating children might have enjoyed including free dental check-ups, check-up reports, free treatment in the clinics of two dental colleges in the study area, education about how to take care of teeth and mouth, and informative booklets to be taken to home.

The consent form was very simple requesting the parents/ guardians to have a tick in either of the boxes for ‘Yes’ or ‘No’ to show whether or not they would like their children to participate in the study. The class teachers in all schools kept on sending reminders to parents till written informed consent forms were received for all children. All parents/ guardians gave a positive consent.

### Oral health education intervention and follow up

The social learning theory [34] formed the theoretical basis of OHE intervention. The DL, TL and PL groups were exposed to a single OHE session after baseline data collection in January 2004 with no further OHE till August 2004. The OHE messages were reinforced on a monthly basis between September 2004 and February 2005 followed by a period of no OHE activity till February 2006. The three groups were subjected to four evaluations during the course of the trial. Evaluation I was conducted immediately after the first education session to observe the effect of the single OHE input on the dependent variables. Evaluation II was performed approximately 6 months after evaluation I to measure the sustainability of the effect resulting from one-time OHE. While evaluation III and evaluation IV were undertaken 6 months and a year after the reinforcement phase of the project respectively to determine the long term impact of the RR-OHE on the outcome variables of the study.

### Data collection methods

The data were collected at baseline and all subsequent evaluations by a structured interview, a pre-tested self-administered questionnaire and oral examination. The study participants were firstly interviewed by a trained dental assistant and then they filled out the questionnaires under the direct supervision of the author and the teachers-in-charge. They finally underwent oral examinations by a trained and calibrated examiner.

The questionnaire consisted of eighteen close-ended questions: twelve concerning oral health knowledge, two related to attitudes towards maintenance of oral hygiene and four pertaining to oral health behavior. The interview comprised of eight oral health behavior questions, one each about adolescents’ practices of buying and sharing various snacks, thoroughness of tooth cleaning and cleaning of cervical areas of teeth, and ascertaining the presence of fluoride in the tooth paste or miswak they used; two about the behavior of adolescents towards keeping the company of peers with bad breath and using betel-nut containing products (BNPs); and one about the adolescents’ role in persuading their peers to avoid/ quit the use of BNPs.

The study participants were examined clinically for dental caries, dental plaque, gingival bleeding on probing and calculus under natural light using a set of sterilized plain mouth mirror and Community Periodontal Index (CPI) probe. They were seated in an ordinary plastic chair. DMFT index, as recommended by WHO, was used to record dental caries in erupted teeth [35]. The buccal/ labial and lingual/ palatal surfaces of two central incisors in the anterior sextants and those of two most posterior teeth in the right and left sextants of the upper and lower dental arches were examined for bleeding on probing and calculus according to WHO criteria for CPI index. Dental plaque was recorded as present or absent on visual and probe examination. Teeth were examined in a sequential order and the findings were recorded by a trained recorder. Re-examination of 5% children in randomly selected schools at baseline and all evaluations yielded Kappa statistics of well above 0.8 for intra-examiner reliability. The parents of all participating children were informed about their children’s oral health status at baseline and all subsequent evaluations.

### Data organization and analyses

The questionnaire and interview data were firstly scored giving ‘1’ to a correct and ‘0’ to an incorrect response to each question. The data were then categorized in seven domains: knowledge about dentition (K-Dent, score: 0–3), knowledge about dental caries (K-Caries, score:0–4), knowledge about gingivitis (K-Gingivitis, score: 0–2), knowledge about causes of oral cancer (K-Cancer, score: 0–3), attitudes towards maintenance of oral hygiene (Attitudes, score: 0–2), behavior towards prevention of gingivitis (OHBG, score:0–6) and preventive behavior about oral cancer (OHBC, score: 0–6). The oral health knowledge and behavior domains were then merged to produce two composite indices: Oral Health Knowledge (OHK-Composite: K-Dent, K-Caries, K-Gingivitis and K-Cancer, score 0–12) and Oral Health Behavior (OHB-Composite: OHBG and OHBC, score 0–12). The data obtained from clinical oral examination were organized to form two indices: DMFT Composite (the number of teeth decayed, missing and filled due to dental caries, score 0–28) and OHS-Composite (the number of sextants of oral cavity free of dental plaque, bleeding on probing and calculus, score 0–12).

All the above indices were treated as outcome variables of the study and subjected to analyses using Generalized Estimating Equations (GEE) with log link function and exchangeable correlation matrix in SPSS 17 program. The cumulative mean index scores for all participants in the three groups (adjusted for gender, type of school, clustering effect and OHE strategy) and the individual mean scores (adjusted for gender, type of school and clustering effect) of OHE strategies at baseline and different evaluations were calculated. These scores were then compared to observe the effect of one-time OHE and RR-OHE. The minimum level of statistical significance set for the study (*p* < 0.05) was adjusted for multiple comparisons by Bonferroni correction in the GEE model. The regression coefficients (effect sizes) obtained from GEE analysis were exponentiated to make them more meaningful [36].

## Results

The study started with 1007 adolescents studying in 24 schools. Sixty five children left their respective schools before the completion of the study while seven children missed one or more evaluations. The total loss of the study participants occurring over the period of the study was about 7.15 % with 9.0, 7.8 and 4.7 % for the DL, TL and PL strategies respectively. No statistically significant differences were found at baseline between the groups of participants who dropped out and those who continued with the study with regard to gender, type of school, oral health knowledge, attitudes, behavior and oral health status. The data of the study subjects who were lost to follow up were therefore excluded from final analysis leaving data of 935 adolescents to be analyzed. Table 1 shows the distribution of the study participants according to OHE strategy, gender and type of school. The mean age of the study participants was 10.2 years (range: 10–11 years) in the beginning of the study.

**Table 1:** Distribution of the study participants (No. & %) according to gender and type of school

School	Gender	DL^a^	TL^b^	PL^c^	Total
Public	Male	81 (35.07)	69 (29.87)	81 (35.07)	231 (48.33)
	Female	73 (29.55)	85 (34.41)	89 (36.03)	247 (51.67)
	Total	154 (32.22)	154 (32.22)	170 (35.56)	478 (51.12)
Private	Male	70 (32.11)	76 (34.86)	72 (33.03)	218 (47.70)
	Female	79 (33.05)	77 (32.22)	83 (34.73)	239 (52.30)
	Total	149 (32.60)	153 (33.48)	155 (33.92)	457 (48.88)
Grand Total		303 (32.41)	307 (32.83)	325 (34.76)	935 (100.0)

^a^ *DL* Dentist-led, ^b^ *TL* Teacher-led, ^c^ *PL* Peer-led strategies of oral health education

### Comparability of the study groups at baseline

Chi-square test and *t*-test were performed to compare the study groups with regard to the number of adolescents, gender, type of school and outcome variables of the study at baseline. A significantly higher percentage of female students had favorable OHB score (*P* < 0.01) and a lower level of dental plaque (*P* < 0.05) compared to their male counterparts. The plaque level of private school pupils was significantly lower than that of public school students (*P* < 0.05). Therefore, ‘gender’ and ‘type of school’ were used as covariates in the GEE model. The study groups did not differ significantly at baseline with regard to other outcome variables.

### The pertinent findings of the study

Table 2 depicts the adjusted cumulative and individual mean scores of the study groups at baseline and different evaluations while Table 3 shows percent change in the mean scores between consecutive evaluations. The scores of the cumulative OHK indices including K-Dent, K-Gingivitis, K-Cancer and OHK-Composite except that of K-Caries index at evaluation I were statistically higher than the corresponding scores at baseline (*p* < 0.05) (Table 3). The scores of all OHK indices at evaluation I except the K-Cancer index score were insignificantly different from those at evaluation II. All these scores exhibited a highly significant improvement from evaluation II to evaluation III (*p* < 0.001) but the K-Dent (*p* < 0.001), K-Gingivitis (*p* < 0.05) and OHK-Composite (*p* < 0.05) scores declined significantly at evaluation IV compared to the scores at evaluation III. The K-Caries and K-Cancer scores, however, did not change statistically between evaluations III and IV.

**Table 2:** Adjusted mean scores^a^ at baseline (BL) and at evaluation I to IV: Effect of repetition & reinforcement on oral health knowledge, behavior & status

			Oral Health Education Strategy		
Domain or Composite Index		Cumulative (*n* = 935)	Dentist-led (*n* = 303)	Teacher-led (*n* = 307)	Peer-led (*n* = 325)
K-Dent (Score: 3)	BL	1.28 (1.16–1.37)	1.25 (1.08–1.43)	1.35 (1.17–1.52)	1.23 (1.05–1.40)
	I	1.63 (1.50–1.71)	1.56 (1.37–1.74)	1.50 (1.31–1.68)	1.82 (1.64–2.00)
	II	1.46 (1.37–1.55)	1.42 (1.24–1.59)	1.40 (1.22–1.58)	1.55 (1.44–1.65)
	III	2.69 (2.57–2.79)	2.82 (2.63–3.00)	2.68 (2.50–2.80)	2.57 (2.41–2.73)
	IV	2.14 (2.03–2.25)	1.95 (1.74–2.16)	2.34 (2.13–2.49)	2.12 (1.90–2.33)
K-Caries (Score: 4)	BL	0.49 (0.41–0.56)	0.42 (0.32–0.52)	0.57 (0.47–0.67)	0.49 (0.40–0.59)
	I	0.73 (0.65–0.80)	0.68 (0.58–0.77)	0.73 (0.64–0.83)	0.79 (0.70–0.89)
	II	0.66 (0.60–0.75)	0.57 (0.50–0.64)	0.68 (0.61–0.76)	0.73 (0.65–0.80)
	III	1.45 (1.37–1.51)	1.42 (1.25–1.59)	1.38 (1.21–1.55)	1.55 (1.38–1.72)
	IV	1.20 (1.14–1.28)	1.13 (1.01–1.24)	1.24 (1.12–1.37)	1.22 (1.10–1.35)
K-Gingivitis (Score: 2)	BL	0.39 (0.33–0.45)	0.42 (0.36–0.48)	0.39 (0.33–0.45)	0.37 (0.31–0.43)
	I	0.62 (0.55–0.67)	0.69 (0.59–0.80)	0.48 (0.38–0.59)	0.68 (0.57–0.78)
	II	0.52 (0.46–0.58)	0.57 (0.48–0.65)	0.44 (0.35–0.53)	0.56 (0.48–0.65)
	III	1.12 (1.06–1.18)	1.20 (1.04–1.37)	1.01 (0.95–1.07)	1.16 (0.99–1.32)
	IV	0.96 (0.90–1.02)	0.97 (0.91–1.03)	0.85 (0.76–0.94)	1.05 (0.93–1.17)
K-Cancer (Score: 3)	BL	0.25 (0.16–0.34)	0.21 (0.15–0.26)	0.29 (0.24–0.35)	0.24 (0.19–0.29)
	I	0.60 (0.52–0.67)	0.73 (0.59–0.86)	0.45 (0.32–0.58)	0.61 (0.48–0.75)
	II	0.37 (0.29–0.44)	0.35 (0.24–0.46)	0.37 (0.30–0.43)	0.38 (0.31–0.46)
	III	1.15 (1.11–1.26)	1.29 (1.09–1.49)	0.94 (0.74–1.13)	1.23 (1.03–1.42)
	IV	1.07 (0.98–1.14)	1.21 (1.04–1.39)	0.82 (0.65–0.99)	1.17 (1.00–1.34)
OHK-Composite (Score: 12)	BL	2.41 (2.15–2.66)	2.29 (2.05–2.54)	2.59 (2.32–2.86)	2.36 (2.12–2.60)
	I	3.57 (3.28–3.86)	3.66 (3.43–3.89)	3.16 (2.84–3.48)	3.90 (3.61–4.19)
	II	3.01 (2.73–3.30)	2.91 (2.64–3.18)	2.89 (2.66–3.12)	3.22 (2.98–3.46)
	III	6.42 (6.16–6.68)	6.69 (6.37–7.02)	6.01 (5.68–6.35)	6.55 (6.25–6.86)
	IV	5.41 (5.14–5.62)	5.33 (4.80–5.87)	5.32 (4.79–5.85)	5.58 (5.05–6.11)
Attitudes (Score: 2)	BL	1.92 (1.90–1.93)	1.95 (1.91–1.98)	1.92 (1.89–1.95)	1.88 (1.85–1.91)
	I	1.95 (1.94–1.97)	1.96 (1.94–1.99)	1.94 (1.92–1.97)	1.95 (1.92–1.97)
	II	1.95 (1.93–1.96)	1.95 (1.92–1.98)	1.96 (1.93–1.98)	1.93 (1.90–1.96)
	III	1.97 (1.96–1.99)	1.97 (1.95–1.99)	1.97 (1.95–1.99)	1.98 (1.96–2.00)
	IV	1.96 (1.94–1.97)	1.97 (1.95–1.99)	1.95 (1.92–1.97)	1.95 (1.93–1.98)
OHB-Gingivitis (Score: 6)	BL	1.64 (1.50–1.79)	1.56 (1.37–1.74)	1.75 (1.57–1.94)	1.62 (1.44–1.80)
	I	2.25 (2.13–2.41)	2.25 (2.07–2.42)	2.14 (1.97–2.31)	2.37 (2.20–2.54)
	II	2.08 (1.95–2.23)	2.01 (1.92–2.13)	2.04 (1.86–2.22)	2.20 (2.11–2.29)
	III	3.88 (3.73–4.02)	3.76 (3.58–3.94)	3.71 (3.45–3.97)	4.18 (4.01–4.36)
	IV	3.83 (3.69–3.97)	3.65 (3.48–3.83)	3.73 (3.55–3.92)	4.10 (3.96–4.25)
OHB-Cancer (Score: 6)	BL	1.81 (1.64–1.97)	1.84 (1.62–2.05)	1.80 (1.62–1.98)	1.78 (1.57–2.00)
	I	2.04 (1.94–2.18)	2.12 (2.02–2.23)	2.00 (1.89–2.11)	1.99 (1.84–2.14)
	II	1.95 (1.88–2.22)	2.03 (1.67–2.17)	1.92 (1.75–2.26)	1.90 (1.91–2.42)
	III	3.77 (3.61–3.93)	3.88 (3.60–4.26)	3.50 (3.12–3.79)	3.94 (3.59–4.26)
	IV	3.61 (3.42–3.77)	3.66 (3.42–3.87)	3.39 (3.18–3.63)	3.79 (3.59–4.04)
OHB-Composite (Score: 12)	BL	3.42 (3.18–3.72)	3.39 (3.09–3.69)	3.59 (3.29–3.89)	3.42 (3.12–3.71)
	I	4.29 (4.00–4.58)	4.37 (4.09–4.65)	4.14 (3.81–4.46)	4.36 (4.09–4.63)
	II	4.03 (3.74–4.32)	4.04 (3.83–4.26)	3.96 (3.73–4.20)	4.10 (3.92–4.28)
	III	7.66 (7.36–7.95)	7.64 (7.35–7.99)	7.21 (6.89–7.53)	8.12 (7.86–8.38)
	IV	7.44 (7.15–7.72)	7.30 (6.97–7.47)	7.14 (6.82–7.47)	7.92 (7.60–8.24)
DMFT-Composite	BL	0.16 (0.11–0.21)	0.13 (0.07–0.19)	0.19 (0.13–0.26)	0.17 (0.11–0.23)
	I	0.16 (0.12–0.21)	0.13 (0.07–0.20)	0.19 (0.13–0.26)	0.17 (0.11–0.23)
	II	0.20 (0.15–0.24)	0.17 (0.10–0.24)	0.22 (0.15–0.28)	0.20 (0.14–0.27)
	III	0.28 (0.23–0.33)	0.25 (0.17–0.33)	0.30 (0.22–0.38)	0.29 (0.21–0.36)
	IV	0.30 (0.25–0.35)	0.31 (0.26–0.36)	0.31 (0.26–0.36)	0.29 (0.21–0.36)
OHS-Composite	BL	3.64 (3.06–4.22)	4.20 (3.52–4.88)	3.49 (2.81–4.16)	3.46 (2.79–4.13)
	I	3.12 (2.55–3.68)	3.59 (3.02–4.16)	3.12 (2.55–3.68)	3.06 (2.47–3.65)
	II	2.63 (2.08–3.19)	3.09 (2.68–3.50)	2.77 (2.36–3.18)	2.56 (2.16–2.96)
	III	4.44 (3.86–5.02)	4.60 (4.15–5.06)	4.56 (4.11–5.01)	4.68 (4.23–5.12)
	IV	4.85 (4.29–5.41)	5.20 (4.66–5.74)	4.86 (4.32–5.39)	5.00 (4.47–5.53)

^a^All scores adjusted for sex, type of school, clustering effect and baseline values using Generalized Estimating Equations; cumulative scores are also adjusted for OHE strategy; Evaluation I: After single OHE session; Evaluation II: Six months after single OHE session; Evaluation III: Six months after RR-OHE; Evaluation IV: Twelve months after RR-OHE; DL group was used as positive control

**Table 3:** Effect of repetition & reinforcement: Percent change in adjusted mean scores at different evaluations

		Cumulative score	DL^a^	TL^b^	PL^c^	Statistical Significance
		(*n* = 935)	(*n* = 303)	(*n* = 307)	(*n* = 325)	
K-Dent (Score: 3)	I vs BL	11.66^*^	10.33^*^	5.00	19.66^**^	I > BL
	I vs II	5.66	4.66	3.33	9.00^*^	I > II
	III vs II	41.00^**^	46.66^**^	42.66^**^	34.00^**^	III > II
	III vs IV	18.33^**^	29.00^**^	11.33^*^	15.00^**^	III > IV
K-Caries (Score: 4)	I vs BL	6.00	6.50	4.00	7.50^*^	I > BL
	I vs II	1.75	2.75	1.25	1.50	NS
	III vs II	19.75^**^	21.25^**^	17.50^**^	20.50^**^	III > II
	III vs IV	6.25	7.25^*^	3.50	8.25^*^	III > IV
K-Gingivitis (Score: 2)	I vs BL	11.50^*^	13.50^*^	4.50	15.50^**^	I > BL
	I vs II	5.00	6.00	2.00	6.00	NS
	III vs II	30.00^**^	31.50^**^	28.50^**^	30.00^**^	III > II
	III vs IV	8.00^*^	11.50^*^	8.00^*^	5.50	III > IV
K-Cancer (Score: 3)	I vs BL	11.66^*^	17.33^**^	5.33	12.33^*^	I > BL
	I vs II	7.66^*^	12.66^*^	2.66	7.66^*^	I > II
	III vs II	26.00^**^	31.33^**^	19.00^**^	28.33^**^	III > II
	III vs IV	2.66	2.66	4.00	2.00	NS
OHK-Composite (Score: 12)	I vs BL	9.66^*^	11.42^*^	4.75	12.83^*^	I > BL
	I vs II	4.66	6.25	2.25	5.66	I > II
	III vs II	28.42^**^	31.50^**^	26.00^**^	27.75^**^	III > II
	III vs V	8.42^*^	11.33^*^	5.75	8.08^*^	III > IV
OHB-Gingivitis (Score: 6)	I vs BL	10.17^*^	11.50^*^	6.50	12.50^*^	I > BL
	I vs II	2.83	4.00	1.67	2.83	NS
	III vs II	30.00^**^	29.17^**^	27.83^**^	33.00^**^	III > II
	III vs IV	0.83	1.83	−0.33	1.33	NS
OHB-Cancer (Score: 6)	I vs BL	3.83	4.67	3.33	3.50	NS
	I vs II	1.50	1.50	1.33	1.50	NS
	III vs II	30.33^**^	30.83^**^	26.33^**^	34.00^**^	III > II
	III vs IV	2.67	3.67	1.83	2.50	NS
OHB-Composite (Score: 12)	I vs BL	7.25^*^	8.17^*^	4.58	7.83^*^	I > BL
	I vs II	2.17	2.75	1.50	2.17	NS
	III vs II	30.25^**^	30.00^**^	27.08^**^	33.50^**^	III > II
	III vs IV	1.83	2.83	0.58	1.67	NS
OHS-Composite (Score: 12)	I vs BL	4.33	5.08	3.08	3.33	NS
	I vs II	4.08	4.17	2.92	4.17	NS
	III vs II	15.08^**^	12.58^**^	14.92^**^	17.67^**^	III > II
	IV vs III	3.42	5.00	2.50	2.67	NS

^a^DL: Dentist-led; ^b^TL: Teacher-led; ^c^PL: Peer-led strategies of oral health education (OHE); Evaluation I: After single OHE session; Evaluation II: Six months after single OHE session; Evaluation III: Six months after RR-OHE; Evaluation IV: Twelve months after RR-OHE;*p < 0.05; **p < 0.001; >:Statistically better than; NS: Statistically insignificant difference; Cumulative scores adjusted for gender, type of school, clustering effect and OHE strategy using GEE; Scores of DL, TL, PL strategies adjusted for gender, type of school and clustering; DL group was used as positive control

It is evident from the results of the study that the sustainability of the knowledge gain resulting from RR-OHE was 168 % higher compared to 24 % sustainability in case of one-time OHE over the period of six-month, when the baseline knowledge was used as a reference in both cases (Table 4). Furthermore the OHK score of adolescents after 12 months of RR-OHE was 126 % greater than their baseline knowledge. It can be observed in Table 4 that the cognitive gain in K-Cancer domain resulting from RR-OHE was at least 4.5 times more likely to sustain over the period of 1 year compared to the baseline knowledge while the retention of knowledge gained by one-time OHE in this domain was only 56 % over a six-month period.

**Table 4:** One-time OHE vs Repeated & Reinforced OHE

	Effect Size			
	Odds Ratio (CI)^a^			
	Evaluation I	Evaluation II	Evaluation III	Evaluation IV
K-Dent	1.27	1.10	2.12	1.69
	(1.18–1.37)	(1.04–1.18)	(2.01–2.24)	(1.60–1.79)
K-Caries	1.50	1.38	2.96	2.49
	(1.33–1.68)	(1.23–1.55)	(2.80–3.15)	(2.21–2.81)
K- Gingivitis	1.57	1.33	2.86	2.44
	(1.36–1.80)	(1.15–1.54)	(2.64–3.10)	(2.25–2.63)
K-Cancer	2.53	1.56	4.79	4.51
	(2.07–3.06)	(1.18–2.05)	(3.58–6.40)	(3.37–6.02)
OHK-Composite	1.49	1.24	2.68	2.26
	(1.35–1.67)	(1.12–1.36)	(2.48–2.90)	(2.09–2.44)
OHB-Gingivitis	1.38	1.27	2.36	2.33
	(1.29–1.48)	(1.18–1.36)	(2.19–2.53)	(2.17–2.50)
OHB Cancer	1.14	1.10	2.09	2.00
	(1.05–1.24)	(1.01–1.20)	(1.93–2.26)	(1.85–2.17)
OHB-Composite	1.26	1.18	2.22	2.17
	(1.18–1.33)	(1.11–1.25)	(2.08–2.37)	(2.03–2.31)
OHS-Composite	0.87	0.75	1.37	1.49
	(0.79–0.97)	(0.66–0.85)	(1.25–1.51)	(1.38-1.60)

Evaluation I: After single OHE session; Evaluation II: Six months after single OHE session; Evaluation III: Six months after RR-OHE; Evaluation IV: Twelve months after RR-OHE; ^a^Baseline score was used as the reference; *CI* 95 % Confidence Interval; All values adjusted for gender, type of school, clustering effect and OHE strategy using GEE; DL group was used as positive control

The cumulative OHB-Gingivitis and OHB-Composite scores of the study participants exhibited a statistically significant improvement at evaluation I compared to the baseline scores (*p* < 0.05) after one-time OHE but the difference between the two scores of the OHB-Cancer index remained statistically non-significant. The scores of the three OHB indices did not change statistically between evaluations I and II, improved significantly between evaluation II to evaluation III (*p* < 0.001) and did not show significant deterioration between evaluations III and IV (Table 4). The study found that the sustainability of the effect of RR-OHE on the OHB of the study participants over six-month period was about 100 % higher (OR:2.22, CI: 2.08–2.37) than that of the effect produced by one-time OHE (OR: 1.18, CI:1.11–1.25) when the effects resulting from two types of education were compared to the baseline OHB score (Table 4). The finding remained almost the same even after 1 year of RR-OHE (OR: 2.17, CI: 2.03–2.31).

**Fig 2:**
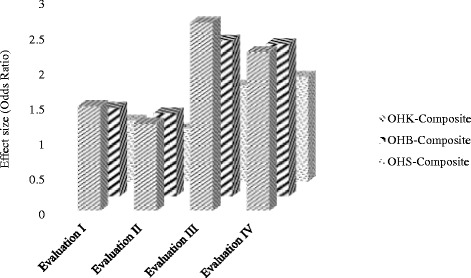
Effect size: One-time vs Repeated & Reinforced OHE

The differences between the cumulative baseline and evaluation I, evaluations I and II, and evaluations III and IV scores of the OHS-Composite index were statistically non-significant at *p* < 0.05. The evaluation II OHS Composite score was, however, significantly lower than the score at evaluation III (*p* < 0.001) (Table 4). The study showed that the adolescents’ OHS-Composite index score, as a result of RR-OHE, remained 37 % and 49 % higher than the respective score at baseline over the period of six-month and 1 year respectively while one-time OHE had virtually no effect on their oral hygiene status (Table 4). The statistical superiority of RR-OHE over one-time OHE in improving OHK, OHB and OHS scores is clearly illustrated in Fig. 2.

The study found that neither one-time OHE nor RR-OHE could have a significant effect on adolescents’ attitudes towards maintenance of oral hygiene as an overwhelming majority (above 95 %) in all three groups already possessed positive attitudes at baseline. Similarly, caries experience of the study subjects and their caries increment remained very low throughout the course of the study reflected by low DMFT scores (Table 2).

The intra-group comparison of the individual scores of the three groups at different evaluations revealed fluctuating trends similar to the ones observed for the cumulative scores of these groups. Additional findings include: The K-Dent and K-Caries scores of the PL group at evaluation I were statistically higher than its corresponding scores at evaluation II and baseline respectively (*p* < 0.05) (Table 4). The differences between baseline and evaluation I scores of all indices related to the oral health knowledge, behavior and oral hygiene status of adolescents in case of TL strategy were statistically insignificant. The OHK-Composite scores of the TL group and the K-Gingivitis scores of the PL group at evaluations III and IV were also statistically insignificant (Table 4).

## Discussion

The study under discussion not only determined the comparative effectiveness of one-time OHE and RR-OHE but also investigated the relevance of these two types of education to different educator-led OHE strategies. The study may be considered important due to its logistic and economic implications on school-based OHE. Repetition in health education involves rehearsal of the same messages again and again. If it happens during the same learning experience, it is called ‘mass repetition’ and if the same information is repeated during different health education sessions, it is known as ‘spaced repetition’ [37]. The existing evidence suggests that spaced repetition, as was practiced in the present study, is better for memory [38]. As reported previously the reinforcement in the present study was provided in the form of an appreciation of a positive oral health behavior by peers [22–24].

A thorough electronic and hand search of the dental literature failed to reveal studies that explored the role of repetition and reinforcement in school-based OHE led by different educators. Also the limited number of studies related to RR-OHE had outcome variables different from the ones included in the present study. The findings of two studies, one by Anaise and Zilkah [16] and the other by Emler et al. [22] are worth comparing with those of the present study. Both studies included repetition and reinforcement as program components and employed Patient Hygiene Performance (PHP) index to measure behavioral change. Anaise and Zilkah reported 47.94 % and 33.56 % behavioral gain in terms of PHP score obtained in the two study groups receiving R-R OHE for 10 months. On the other hand the PHP scores of the groups not exposed to the R-R OHE deteriorated significantly at 2-month and 12-month follow-up examinations. Similarly the PHP score in the Emler et al. study [22] showed a positive percent change of 31.53 % in the PHP score from baseline to the evaluation conducted 8 weeks after R-R OHE. In the study under discussion the percent gain observed in oral hygiene behavior after 6 months of R-R OHE (30 %) was almost comparable to the findings of the two former studies. The finding of the present study, however, seems to be more valid and reliable than those of the aforementioned studies due to its larger group size and the adjustment of percent gain in oral health behavior for clustering effect, confounding variables and multiple statistical comparisons. Furthermore, the present study unlike the aforementioned studies involved estimation of the effect of one-time OHE and that of R-R OHE for the same group of participants.

Convincing evidence for the effectiveness of RR-OHE also came from a cluster randomized controlled trial testing the effectiveness of a school- based DHE program in the UK by Redmond et al. [23]. The trial found that the group of children receiving DHE sessions over the period of 12 months (early intervention group) performed statistically better than the group receiving the same DHE program for 6 months (late intervention group) with regard to oral health knowledge, behavior and plaque score. In that trial, 76–80 % of children in both early and late intervention groups reported brushing their teeth in the morning and evening after 6 months of RR-OHE which is comparable with the finding of the present trial where about 73 % of adolescents reported twice daily tooth brushing 6 months after RR-OHE.

In the present study one-time OHE by dentist and peer leaders resulted in a significant improvement in oral health knowledge and behavior of adolescents but one-time teacher-led OHE failed to produce a statistically significant improvement in any of the indices related to oral health knowledge and behavior. However, the oral health knowledge of the study participants in the dentist- and peer- educated groups deteriorated to a significant extent at 12-month follow up of R-R OHE but that of the teacher-educated group was sustained. The adolescents’ behavior towards prevention of oral cancer and their oral hygiene status did not improve significantly as a result of one-time OHE in all three education strategies. On the contrary the oral hygiene behavior and status of the study participants showed a marked improvement at 6-month follow up of RR-OHE irrespective of the OHE strategy used. During further 6-month follow up of RR-OHE the adolescents’ oral hygiene behavior exhibited a very little deterioration while their oral hygiene status showed a continuous improvement. Moreover, the adolescents’ knowledge and behavior towards prevention of oral cancer, after exhibiting a highly significant enhancement at 6- month follow up of RR-OHE, experienced an insignificant decline at 12-month evaluation. These findings are very encouraging from public health point of view especially in the developing countries where poor oral hygiene [30, 39–44] and betel-nut chewing habit [45–48] are threatening the oral and general health status of a significant proportion of adolescents.

The results of the study not only reconfirmed the significance of repetition and reinforcement in school-based OHE but also highlighted the important role the trained teachers and peers can play in RR-OHE and hence in enhancing the cost-effectiveness, sustainability and availability of school-based OHE.

### Recommendations

The study, being an efficacy trial of 2 years’ duration, enjoyed the controlled and favorable conditions for involvement of teachers and peer leaders in oral health education. A large scale community trial is recommended to confirm the findings of the study and to ascertain the fidelity of implementation of the oral health education strategies in question under real life conditions.

The repeated use of the same questionnaire five times during the project might have led to boredom on the part of the study subjects. The use of diversified data collection techniques is recommended for future research on the subject.

As the present study did not perform the cost-effectiveness and cost-benefit analyses, it is unclear whether the yield would justify the cost incurred in implementing any of the OHE strategies evaluated in the study under daily life conditions. These analyses should, therefore, make an integral part of future school-based trials of OHE interventions.

## Conclusions

The repetition and reinforcement play a key role in the success of a school-based OHE program no matter whether it is led by dentists, teachers or peer leaders. The findings of the study suggest a complimentary role of trained teachers and peers who can act as all-time available experts in the school system to periodically repeat and reinforce OHE messages.

## Abbreviations

Attitudes: Attitudes towards maintenance of oral hygiene
BNPs: Betel-nut containing products
CL: Control
CPI: Community Periodontal Index
DHE: Dental health education
DL: Dentist-led
DMFT: Decayed, missing and filled teeth due to dental caries
K-Cancer: Knowledge about causes of oral cancer
K-Caries: Knowledge about dental caries
K-Dent: Knowledge about dentition
K-Gingivitis: Knowledge about gingivitis
OHB: Oral Health Behavior
OHBC: Preventive behavior about oral cancer
OHBG: Behavior towards prevention of gingivitis
OHE: Oral health education
OHK: Oral Health Knowledge
OHS: Oral Hygiene Status
PL: Peer-led
RR-OHE: Repeated and reinforced oral health education
TCPs: Tobacco-containing products
TL: Teacher-led

## References

[1] Brukiene V, Aleksejuniene J (2010). Theory-based oral health education in adolescents. Stomatol Baltic Dent Max J.

[2] Hartley J (1998). Learning and studying: A research perspective.

[3] Green LW, Kreuter MN (2005). Health promotion planning: An educational and ecological approach.

[4] Gilbert G, Sawyer R, McNeill EB (2011). Health education: Creating strategies for school and community health.

[5] Sprod AJ, Anderson R, Treasure ET (1996). Effective oral health promotion: literature review.

[6] Watt RG, Marinho VC (2005). Does oral health promotion improve oral hygiene and gingival health?. Periodontol 2000.

[7] Hausen H (2005). Oral health promotion reduces plaque and gingival bleeding in the short term. Evid Based Dent.

[8] Shiller WR, Dittmer JC (1968). An evaluation of some current oral hygiene motivation methods. J Periodontol.

[9] Podshadley AG, Schweikle ES (1970). The effectiveness of two educational programs in changing the performance of oral hygiene by elementary school children. J Public Health Dent.

[10] Podshadley AG, Shannon JH (1970). Oral hygiene performance of elementary school children following dental health education. ASDC J Dent Child.

[11] Gjermo P (1972). Audio-visual motivation and oral hygiene instruction. The effect upon gingival status and oral cleanliness in 15 years old children. Odontol Revy.

[12] Ivanovic M, Lekic P (1996). Transient effect of a short-term educational programme without prophylaxis on control of plaque and gingival inflammation in school children. J Clin Periodontol.

[13] Goel P, Sehgal M, Mittal R (2005). Evaluating the effectiveness of school-based dental health education program among children of different socio-economic groups. J Indian Soc Pedod Prev Dent.

[14] Flanders RA (1987). Effectiveness of dental health education programs in schools. JADA.

[15] Tolvanen M, Lahti S, Poutanen R, Seppä L, Hausen H (2010). Children’s oral health-related behaviors: individual stability and stage transitions. Community Dent Oral Epidemiol.

[16] Anaise JZ, Zilkah E (1976). Effectiveness of a dental education program on oral cleanliness of schoolchildren in Israel. Community Dent Oral Epidemiol.

[17] Toassi RF, Petry PC (2002). [Motivation on plaque control and gingival bleeding in school children]. Rev Saude Publica.

[18] Shenoy RP, Sequeira PS (2010). Effectiveness of a school dental education program in improving oral health knowledge and oral hygiene practices and status of 12- to 13-year-old school children. Indian J Dent Res.

[19] D’Cruz AM, Aradhya S (2013). Impact of oral health education on oral hygiene knowledge, practices, plaque control and gingival health of 13- to 15-year-old school children in Bangalore city. Int J Dent Hyg.

[20] Craft M, Croucher R, Dickinson J, James M, Clements M, Rodgers AI (1984). Natural Nashers: a programme of dental health education for adolescents in schools. Int Dent J.

[21] Ferrazzano GF, Cantile T, Sangianantoni G, Ingenito A (2008). Effectiveness of a motivation method on the oral hygiene of children. Eur J Paediatr Dent.

[22] Emler BF, Windchy AM, Zaino SW, Feldman SM, Scheetz JP (1980). The value of repetition and reinforcement in improving oral hygiene performance. J Periodontol.

[23] Redmond CA, Blinkhorn FA, Kay EJ, Davies RM, Worthington HV, Blinkhorn AS (1999). A cluster randomized controlled trial testing the effectiveness of a school-based dental health education program for adolescents. J Public Health Dent.

[24] Rodrigues JA, dos Santos PA, Baseggio W, Corona SA, Palma-Dibb RG, Garcia PP (2009). Oral hygiene indirect instruction and periodic reinforcements: effects on index plaque in schoolchildren. J Clin Pediatr Dent.

[25] Baab DA, Weinstein P (1983). Oral Health instruction using a self-inspection plaque index. Community Dent Oral Epidemiol.

[26] Nowjack-Raymer R, Ainamo J, Suomi JD, Kingman A, Driscoll WS, Brown LJ (1995). Improved periodontal status through self-assessment. A 2-year longitudinal study in teenagers. J Clin Periodontol.

[27] Kallio P, Uutela A, Nordblad A, Alvesalo I, Murtomaa H, Croucher R (1997). Self-assessed bleeding and plaque as methods for improving gingival health in adolescents. Int Dent J.

[28] Haleem A, Siddiqui MI, Khan AA (2012). School-based strategies for oral health education of adolescents-a cluster randomized controlled trial. BMC Oral Health.

[29] Ali Z (2012). Violence in Karachi: Is it political, ethnic or religious conflict?. Pakistaniaat (J Pak Studies).

[30] Whitley E, Ball J (2002). Statistic review 4: Sample size calculations. Crit Care.

[31] Hemming K, Girling AJ, Sitch AJ, Marsh J, Lilford RJ (2011). Sample size calculations for cluster randomised controlled trials with a fixed number of clusters. BMC Med Res Methodol.

[32] Khan AA, Sharea I, Ayma S, Ambreena Q, Inayatullah P, Sofia S (2004). Oral health in Pakistan: A situation analysis. Dev Dent.

[33] Kirkwood B, Sterne J (2001). Essentials of Medical Statistics.

[34] Parcel GS, Baranowski T (1981). Social learning theory and health education. Health Educ.

[35] World Health Organization (1997). Oral health surveys: basic methods.

[36] Ballinger GA (2004). Using generalized estimating equations for longitudinal data analysis. Organ Res Methods.

[37] Lowenstein AJ, Foord L, Romano JC (2009). Teaching strategies for health education and health promotion. Working with patients, families and communities.

[38] Seabrook R, Brown GD, Solity JE (2005). Distributed and massed practice: from laboratory to class room. Appl Cogn Psychol.

[39] Prasai Dixit L, Shakya A, Shrestha M, Shrestha A (2013). Dental caries prevalence, oral health knowledge and practice among indigenous Chepang school children of Nepal. BMC Oral Health.

[40] Carneiro LC, Kabulwa MN (2012). Dental Caries, and Supragingival Plaque and Calculus among Students, Tanga, Tanzania. ISRN Dent.

[41] Sarwar AFM, Kabir MH, Rahman AFMM, Haque A, Kasem MA, Ahmad SA (2012). Oral hygiene practice among the primary school children in selected rural areas of Bangladesh. J Dhaka National Med Coll Hosp.

[42] Jürgensen N, Petersen PE (2011). Oral health behaviour of urban and semi-urban schoolchildren in the Lao PDR. Community Dent Health.

[43] Muwazi LM, Rwenyonyi CM, Tirwomwe FJ, Ssali C, Kasangaki A, Nkamba ME (2005). Prevalence of oral diseases/conditions in Uganda. Afr Health Sci.

[44] David J, Wang NJ, Astrøm AN, Kuriakose S (2005). Dental caries and associated factors in 12-year-old schoolchildren in Thiruvananthapuram, Kerala. India Int J Paediatr Dent.

[45] Oakley E, Demaine L, Warnakulasuriya S (2005). Areca (betel) nut chewing habit among high-school children in the Commonwealth of the Northern Mariana Islands (Micronesia). Bull World Health Organ.

[46] Prabhu NT, Warnakulasuriya K, Gelbier S, Robinson PG (2001). Betel quid chewing among Bangladeshi adolescents living in East London. Int J Paediatr Dent.

[47] Hingorjo MR, Jaleel F, Mehdi A (2010). Oral health aspects In primary school children of three major cities of Pakistan. JPDA.

[48] Shah S, Qureshi R, Azam I (2009). Is Chaalia/Pan Masala harmful for health? Practices and knowledge of children of schools in Mahmoodabad and Chanesar Goth, Karachi. J Pak Med Assoc.

